# p66Shc deficiency in CLL cells enhances PD-L1 expression and suppresses immune synapse formation

**DOI:** 10.3389/fcell.2024.1297116

**Published:** 2024-01-23

**Authors:** Ludovica Lopresti, Nagaja Capitani, Vanessa Tatangelo, Carmela Tangredi, Gioia Boncompagni, Federica Frezzato, Andrea Visentin, Giuseppe Marotta, Sara Ciofini, Alessandro Gozzetti, Monica Bocchia, Livio Trentin, Cosima T. Baldari, Laura Patrussi

**Affiliations:** ^1^ Department of Life Sciences, University of Siena, Siena, Italy; ^2^ Hematology and Clinical Immunology Unit, Department of Medicine, University of Padua, Padua, Italy; ^3^ Stem Cell Transplant and Cellular Therapy Unit, University Hospital, Siena, Italy; ^4^ Department of Medical Science, Surgery and Neuroscience, University of Siena, Siena, Italy

**Keywords:** PD-L1, immune checkpoint, CLL, p66Shc, immune synapse, ROS

## Abstract

**Introduction:** Escape from immunosurveillance is a hallmark of chronic lymphocytic leukemia (CLL) cells. In the protective niche of lymphoid organs, leukemic cells suppress the ability of T lymphocytes to form the immune synapse (IS), thereby hampering T-cell mediated anti-tumoral activities. By binding its cognate receptor PD-1 at the surface of T lymphocytes, the inhibitory ligand PD-L1, which is overexpressed in CLL cells, mediates the T-cell suppressive activities of CLL cells. However, the molecular mechanism underlying PD-L1 overexpression in CLL cells remains unknown. We have previously reported a defective expression of the pro-apoptotic and pro-oxidant adaptor p66Shc in CLL cells, which is causally related to an impairment in intracellular reactive oxygen species (ROS) production and to the activation of the ROS-sensitive transcription factor NF-κB. The fact that PD-L1 expression is regulated by NF-κB suggests a mechanistic relationship between p66Shc deficiency and PD-L1 overexpression in CLL cells.

**Methods:** 62 treatment-naive CLL patients and 43 healthy donors were included in this study. PD-L1 and p66Shc expression was quantified in B cells by flow cytometry and qRT-PCR. IS architecture and local signaling was assessed by flow cytometry and confocal microscopy. CD8+ cell killing activity was assessed by flow cytometry.

**Results:** Here we show that residual p66Shc expression in leukemic cells isolated both from CLL patients and from the CLL mouse model Eμ-TCL1 inversely correlated with PD-L1 expression. We also show that the PD-L1 increase prevented leukemic cells from forming ISs with T lymphocytes. Reconstitution of p66Shc, but not of a ROS-defective mutant, in both CLL cells and the CLL-derived cell line MEC-1, enhanced intracellular ROS and decreased PD-L1 expression. Similar results were obtained following treatment of CLL cells with H_2_O_2_ as exogenous source of ROS, that normalized PD-L1 expression and recovered IS formation.

**Discussion:** Our data provide direct evidence that the p66Shc-deficiency-related ROS depletion in CLL cells concurs to enhance PD-L1 expression and provides a mechanistic basis for the suppression of T cell-mediated anti-tumoral functions in the immunosuppressive lymphoid niche.

## 1 Introduction

The efficient evasion of anti-tumor immune responses is a hallmark of chronic lymphocytic leukemia (CLL) cells (Capitani et al., 2021). In the protective niche of secondary lymphoid organs and bone marrow, leukemic cells contribute to the establishment of an immunosuppressive microenvironment by secreting soluble factors, such as interleukin (IL)-10, that promote regulatory T cell development and suppression of anti-tumoral effector T cell functions (ten Hacken and Burger, 2016; Beyer et al., 2005). Additionally, CLL cells negatively modulate T cell activity through direct contact mediated by inhibitory surface receptor/ligand pairs. PD-L1, which is upregulated at the surface of CLL cells (Ramsay et al., 2012; Grzywnowicz et al., 2015), interacts with its receptor PD-1 whose expression, transiently enhanced during the physiological activation processes, is aberrantly upregulated on effector T cells in the stromal CLL niche (Riches et al., 2013), thus potentiating the PD1/PD-L1 signaling axis. PD-L1 has a major negative impact on the assembly of a functional immune synapse (IS), the signaling platform that T cells assemble at the interface with cognate antigen presenting cells (APC) by triggering the polarization of receptors, adhesion molecules, cytoskeletal components and secretory machinery toward the T cell/APC contact area (Fooksman et al., 2010; Alarcón et al., 2011; Cassioli and Baldari, 2022). The formation of a stable IS architecture, which is a key prerequisite for the execution of T cell effector functions (Dustin and Choudhuri, 2016), is consistently disabled in the CLL context. This occurs at least in part as a result of PD-L1-dependent PD-1 triggering (Ramsay et al., 2012) which leads to the recruitment of the phosphatase SHP-2 close to the T Cell Receptor (TCR) (Saunders et al., 2005), thereby shutting down antigen-dependent signaling (Arasanz et al., 2017). By reactivating anti-tumor T cell functions, immune checkpoint inhibitor-based immunotherapies targeting the PD-1/PD-L1 axis showed promising results in clinical trials enrolling Hodgkin lymphoma, follicular lymphoma and diffuse large B-cell lymphoma patients (Kline et al., 2020), witnessing to the key role of this inhibitory signaling axis in the pathogenesis of hematological malignancies. Of note, the modest efficacy observed for CLL patients, which was limited to patients with the aggressive Richter transformation who experienced increased PD-1 expression during treatment (Ding et al., 2017; Kline et al., 2020; Ioannou et al., 2021), together with the large variability of both PD-1 and PD-L1 expression in CLL patients, underlie the low effectiveness of neutralizing antibody-based therapies (Brusa et al., 2013; Ding et al., 2017) and highlight the need of alternative therapeutic approaches to target this signaling axis in CLL.

In B lymphocytes, the adaptor protein p66Shc negatively regulates cell survival by modulating the expression of apoptosis-regulating proteins belonging to the Bcl-2 family and inhibiting the activation of the pro-survival kinase Akt (Capitani et al., 2010). Additionally, it participates in signaling pathways linking oxidative stress to apoptosis (Migliaccio et al., 1999; Patrussi et al., 2020). By interrupting the mitochondrial respiratory chain through cytochrome-c binding and oxidation, p66Shc promotes ROS production and activates the apoptotic cascade (Giorgio et al., 2005; Pellegrini et al., 2007). In CLL cells a profound p66Shc decrease, which is most pronounced in leukemic cells with unmutated *IGHV* genes (U-CLL), has been related to enhanced leukemic cell survival and drug resistance (Capitani et al., 2010; Patrussi et al., 2019). The resulting decrease in intracellular ROS promotes the expression of the chemokine receptors CCR2, CXCR3 and CCR7, which are implicated in CLL cell homing to lymphoid and non-lymphoid organs, as a result of enhanced activity of the transcription factor NF-κB (Capitani et al., 2012; Tatangelo et al., 2022). The p66Shc defect also impacts on the expression of the egress receptor sphingosine-1-phosphate receptor 1 (S1PR1) in U-CLL cells, thereby further promoting leukemic cell entrapment in the pro-survival tumoral niche (Capitani et al., 2012).

The implication of p66Shc deficiency in CLL onset and development is witnessed by the rapid development of an aggressive form of the disease in Eμ-TCL1/p66Shc^−/−^ mice, generated by introducing a null p66Shc allele into the Eµ-TCL1 mouse model of human CLL (Bichi et al., 2002; Patrussi et al., 2019). However, the potential impact of the p66Shc defect on immunosuppressive activities of leukemic cells remains to be clarified. Here, we have addressed the outcome of p66Shc deficiency on the ability of CLL cells to form ISs with T lymphocytes. We show that p66Shc deficiency leads to enhanced expression of PD-L1, which in turn contributes to suppress IS formation with Jurkat T cells, a defect that was reversed by restoring p66Shc expression. Furthermore, we demonstrate that the p66Shc defect in CLL cells impinges on PD-L1 overexpression and IS suppression by downregulating the intracellular ROS content. Altogether, our data provide direct evidence that ROS depletion caused by p66Shc deficiency concurs to the formation of a T cell-suppressive tumor microenvironment in CLL.

## 2 Materials and methods

### 2.1 CLL patients, healthy donors and mice

Peripheral blood samples were collected from 62 treatment-naive CLL patients. CLL diagnosis and mutational *IGHV* status were assessed as reported (Hallek et al., 2008; Patrussi et al., 2019; Visentin et al., 2019). Healthy B cells obtained from 43 buffy coats were used as healthy population controls. Human B cells were purified by negative selection using RosetteSep B-cell enrichment Cocktail (StemCell Technologies, Vancouver, Canada) followed by density gradient centrifugation on Lympholite (Cedarlane Laboratories, The Netherlands).

Mice included Eμ-TCL1 and Eμ-TCL1/p66Shc^−/−^ mice (Bichi et al., 2002; Patrussi et al., 2019), and parental C57BL/6J mice. Disease development and overt leukemia achievement were assessed as reported (Patrussi et al., 2019). Leukemic cells were purified from age-matched 9–12-month-old EµTCL1 or Eμ-TCL1/p66Shc^−/−^ mice by immunomagnetic activated cell-sorting (MACS, Miltenyi Biotech). Splenic B cells were purified from C57BL/6 mice as reported (Patrussi et al., 2018).

### 2.2 Cell lines and transfections

Stable transfectants generated using the CLL-derived B-cell line MEC-1 and expressing human full-length p66Shc or the p66ShcQQ mutant were previously described (Stacchini et al., 1999; Capitani et al., 2012). CLL cells were transiently transfected with 5 μg pcDNA3 or p66Shc-encoding pcDNA3 or p66ShcQQ pBabe vectors/sample using the Human B-cell Nucleofector Kit (Amaxa Biosystems, Cologne, Germany) as described (Patrussi et al., 2019). Assays were carried out after 24/48 h. For NF-AT translocation assays, Jurkat T cells were transiently transfected with the plasmid pEGFP/NFAT-1D (Plyte et al., 2001) by electroporation.

### 2.3 Purification and activation of CD8^+^ cells

CD8^+^ cells were isolated from peripheral blood of healthy donors by negative selection using the RosetteSep Human CD8^+^ T Cell Enrichment Cocktail (StemCell Technologies, #15063), following the manufacturer’s instructions. On the same day (day 0), cells were stimulated in RPMI-HEPES medium (1×10^6^/mL) (Merck, #R7388) supplemented with 10% BCS (GE Healthcare HyClone, #SH30072.03), 1% MEM nonessential amino acids (MEM NEAA; #11140050), and 50 U/mL recombinant human IL-2 with Dynabeads™ Human T-activator CD3/CD28. 48 h after activation (day 2), beads were removed and CTLs were collected. For cytotoxicity assays, CTLs were expanded in RPMI-HEPES supplemented with 10% BCS, 1% MEM NEAA, and 50 U/mL recombinant human IL-2 for additional 3 days, then further expanded for 2 days and collected at day 7 (Onnis et al., 2023).

### 2.4 Cell treatments, ROS measurements, flow cytometry and immunoblot

Freshly isolated healthy and leukemic human B cells, and MEC-1 transfectants were treated with 100 μM H_2_O_2_ (Merck, #H1009). After 6 h, intracellular ROS were measured by flow cytometry in cells labeled for 30 min at 37°C with 5 μM CM-H_2_DCFDA (Capitani et al., 2012). H_2_O_2_ dose and treatment time were previously determined (Tatangelo et al., 2022). Leukemic cell and MEC-1 transfectants were treated with 1 μM IT-901 (R&D Systems, #5846) for 24 h. Control samples were treated with DMSO. Phospho-NF-κB was quantified after 30 min by flow cytometry in cells fixed and permeabilized using Fixation (Biolegend, #420801) and Permeabilization (Biolegend, #421002) buffers and stained with anti-human phospho-NF-κB p65 (Ser536) (Cell Signaling Technology, #3031) and Alexa Fluor-647 anti-rabbit secondary antibodies (Thermo Fisher Scientific, #A21236). Surface expression of PD-L1 was assessed by flow cytometry. MEC transfectants and B cells purified from peripheral blood of CLL patients and healthy donors were stained with anti-PD-L1 antibodies (Invitrogen, #PA5-23043) and Alexa Fluor 647- labelled anti-rabbit secondary antibodies (Thermo Fisher Scientific, #A21236). Single-cell suspensions obtained from mouse spleens were depleted of erythrocytes by hypotonic lysis and incubated with mouse Fc-block for 15 min at 4°C. Murine B cells were stained as above and subjected to flow cytometry using Guava Easy-Cyte cytometer (Merck Millipore) as described (Patrussi et al., 2015). Antibodies and reagents are listed in Supplementary Table S1.

Cells (2×10^6^/sample) were lysed in 1% (v/v) Triton X-100 in 20 mM Tris-HCl pH 8, 150 mM NaCl in the presence of Protease Inhibitor Cocktail (Calbiochem^®^, #539–134) and 0.2 mg/mL sodium orthovanadate (Merck, #S6508), for 5 min on ice. Protein extracts were quantified by Quantum Protein Assay Kit (Euroclone, #EMP014500) and denatured in 4× Bolt™ LDS Sample Buffer (Thermo Fisher Scientific, #B0007) supplemented with 10× Bolt™ Sample Reducing Buffer (Thermo Fisher Scientific, #B009) for 5 min at 100°C. Proteins (10 μg) were resolved by SDS-PAGE (Life Technologies, #NW04120BOX) and transferred to nitrocellulose (GE Healthcare, #9004–70-0) as previously described (Patrussi et al., 2018). Membranes were incubated with primary antibodies for 3 h at room temperature or overnight at 4°C. Immunoblots were carried out using anti-Shc (Merck Millipore, #06–203), anti-PD-L1 (Invitrogen, #PA5-20343) and anti-actin (Millipore, #MAB1501) primary antibodies. Secondary peroxidase-labeled anti-mouse (#115–035–146) and anti-rabbit (#111–035-003) antibodies were from Jackson Immuno-Research (Supplementary Table S1). Labeled antibodies were detected using ECL kit (SuperSignal^®^ West Pico Chemiluminescent Substrate, Thermo Fisher Scientific #34578), and immunoblots were digitally acquired and analyzed using Alliance Q9-Atom chemiluminescence imaging system (Uvitec).

### 2.5 RNA purification and qRT-PCR

RNA was extracted and retrotranscribed as described (Patrussi et al., 2018). Quantitative real-time PCR (qRT-PCR) was performed on 96-well optical PCR plates (Sarstedt) using SsoFast™ EvaGreen^®^ Supermix (Bio-Rad, #1725204) using the CFX96 Real-Time system (Bio-Rad Laboratories). Primers used for amplification are listed in Supplementary Table S2. Results were processed and analyzed as described (Patrussi et al., 2018). The relative gene transcript abundance was determined on triplicate samples using the ddCt method and normalized to either HPRT1 (human-derived samples) or GAPDH (mouse-derived samples).

### 2.6 IS formation and immunofluorescence

Purified healthy or CLL B cells (0.4×10^6^ cells/100 μL) were loaded with 10 μg/mL staphylococcal enterotoxin E (SEE; Toxin Technologies, #ET404) for 1 h and labelled with 10 μM Cell Tracker Blue (Thermo Fisher Scientific, #C2110) for the last 20 min of incubation. SEE was used for Jurkat cells that express TCR Vβ8. For ISs with CTLs, healthy or CLL B cells (0.4×10^6^ cells/100 μL) were loaded with 10 μg/mL Staphylococcal SAg A (SEA; Toxin Technologies, #AT101), B (SEB; Toxin Technologies, #BT202) and E (SEE; Toxin Technologies, #ET404) for 2 h, to broadly cover the TCR Vβ repertoire, and labelled with 10 μM Cell Tracker Blue (Thermo Fisher Scientific, #C2110) for the last 20 min of incubation. SAgs-loaded B cells were washed twice, mixed with either Jurkat cells (2:1) or CTLs (2:1) and incubated for 15 min at 37°C. When required, 5 μg/mL anti-PD-L1 neutralizing mAbs (EliteRmab, #10084-R639) were added to the medium during conjugate formation. Samples were seeded on microscope slides (Thermo Fisher Scientific, #X2XER208B) coated with poly-L-lysine (Merck, #P1274) and allowed to adhere for 15 min at 37°C. Then, samples were fixed for 10 min in methanol at −20°C (for CD3ζ and p-Tyr staining) or for 15 min with 4% paraformaldehyde/PBS at room temperature (for F-actin staining with phalloidin) and permeabilized with 0.1% Triton in 1% BSA PBS. Cells were stained with primary antibodies overnight at 4°C, washed with PBS, incubated for 45 min at room temperature with Alexa Fluor 488- and 555-labeled secondary antibodies and mounted with 90% glycerol/PBS (Cassioli et al., 2021). For phospho-ERK1/2 quantification, Jurkat T cells were conjugated with B cell for 15 min at 37°C, subjected to strong pipetting to disrupt conjugates, fixed and permeabilized using Fixation (Biolegend, #420801) and Permeabilization (Biolegend, #421002) buffers and stained with PE anti-human CD3ε antibody (BioLegend, #317308) and anti-phospho-ERK1/2 (Thr202/Tyr204; Cell Signaling, #9101) antibodies. The percentage of phospho-ERK1/2^+^ cells was quantified by flow cytometry on CD3^+^ positive cells. For NF-AT nuclear translocation assays, Jurkat T cells transfected with the plasmid pEGFP/NFAT-1D (Plyte et al., 2001) were cultured at 37°C for 24 h, then mixed with SEE-loaded B cells for 1 h at 37°C. Jurkat T cell transfectants either unstimulated (ns) or stimulated for 30 min at 37°C with 1 μg/mL A23187 (A23) were used as negative and positive controls, respectively (Pacini et al., 2004). Cells were then allowed to adhere to poly-L-lysine-coated slides for 15 min, fixed with 4% paraformaldehyde/PBS at room temperature, permeabilized and stained with anti-GFP primary antibodies (Invitrogen, #A11120) and Alexa Fluor 488-labeled anti mouse secondary antibodies (Thermo Fisher Scientific, #A11001). Confocal microscopy was carried out on a Zeiss LSM700 (Carl Zeiss, Jena, Germany) microscope using a 63×/1.40 oil immersion objective. Images were acquired with pinholes opened to obtain 0.8 μm-tick sections. Images were processed with Zen 2009 image software (Carl Zeiss, Jena, Germany). Immunofluorescence analyses were performed using ImageJ (RRID:SCR_003070). Scoring of conjugates for accumulation of CD3ζ, p-Tyr or F-actin at the IS was performed as reported (Cassioli et al., 2021; Onnis et al., 2023). Antibodies used for immunofluorescence microscopy are listed in Supplementary Table S1.

### 2.7 Cytotoxicity assays

B cells (0.025×10^6^) were pulsed with 2 μg/mL SAgs for 1 h in serum-free AIMV medium. Unpulsed B cells were used as negative control. CTLs collected at day 7 were stained with 1.5 μM carboxyfluorescein diacetate succinimidyl ester (CFSE; Thermo Fisher Scientific, #C34554) for 8 min at room temperature in PBS and then added to B cells at 1:10 B cell:CTL ratio in 50 μL AIMV (Gibco, #12055-091) medium and incubated at 37°C for 4 h to evaluate target cell killing. When required, 5 μg/mL anti-PD-L1 neutralizing mAbs were added to the medium during conjugate formation. Cells were then diluted to 200 μL with cold PBS and acquired using a GUAVA flow cytometer. Propidium iodide (PI, Sigma, #537059) was added before each acquisition to the final concentration of 20 μg/mL. Cytotoxicity (% target cell lysis) was calculated as follows: CFSE^−^PI^+^ cells–CFSE^−^PI^+^ cells in control sample) × 100.

### 2.8 Study approval

Written informed consent was received from CLL patients and healthy donors prior to inclusion in the study according to the Declaration of Helsinki. Experiments were approved by the local Ethics Committee.

### 2.9 Statistical analyses

Each experiment was performed ≥3 independent times. One-way ANOVA tests with post-hoc Tukey correction and multiple comparisons were used to compare multiple groups. Mann-Whitney rank-sum and Paired t tests were performed to determine the significance of the differences between two groups. One-sample Wilcoxon tests were used where samples were compared to a known standard value. Power and sample size estimations were performed using G*Power (RRID:SCR_013726). Statistical analyses were performed using GraphPad Prism (RRID:SCR_002798). Statistical significance was defined as: ****p ≤ 0.0001; ***p ≤ 0.001; **p ≤ 0.01; *p ≤ 0.05.

## 3 Results

### 3.1 The p66Shc expression defect in CLL cells impinges on their ability to form ISs with Jurkat T cells

**FIGURE 1:**
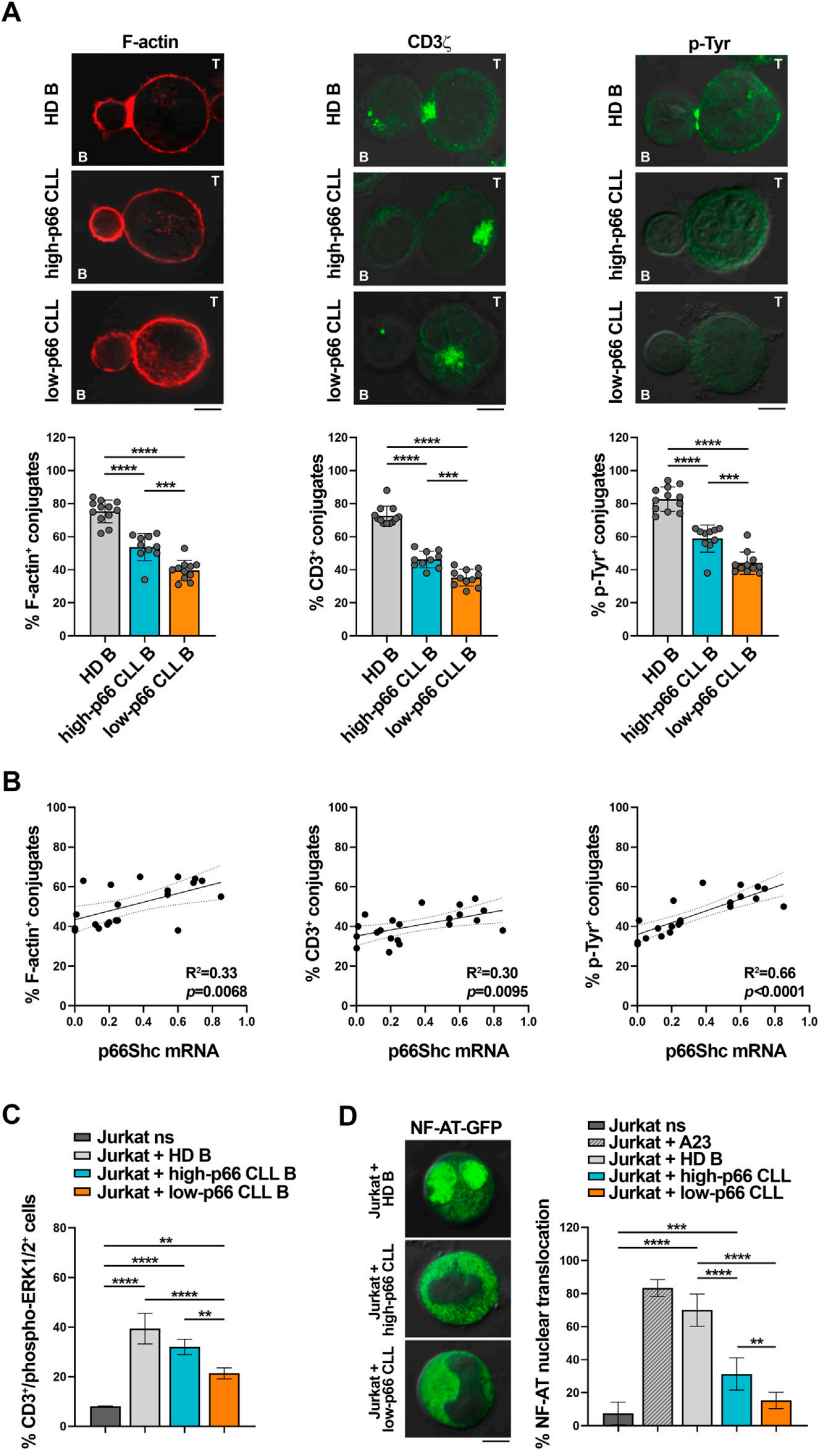
CLL cells with low p66Shc expression suppress the ability of Jurkat T cells to form ISs. **(A)**. Immunofluorescence analysis of F-actin, CD3ζ and p-Tyr in Jurkat T cells mixed with B cells purified from buffy coats of healthy donors (HD B) or peripheral blood of CLL patients with p66Shc expression above (high-p66) or below (low-p66) threshold (0.25 median ddCt value, see Supplementary Figure S1A), pulsed with SEE and incubated for 15 min at 37°C. Data are expressed as % of 15-min SEE-specific conjugates harboring staining at the IS (≥50 cells/sample, n independent experiments≥3). One-way ANOVA test; *p ≤ 0.05; **p ≤ 0.01; ***p ≤ 0.001; ****p ≤ 0.00001. Representative images (medial optical sections) of the Jurkat T cell/B cell conjugates are shown. Scale bar, 5 μm. **(B)**. Correlation between F-actin^+^, CD3ζ^+^ and p-Tyr^+^ Jurkat/B conjugates shown in **(A)** and mRNA levels of p66Shc in CLL cells used in the respective IS experiments (n = 21). Simple linear regression; *p* ≤ 0.05 statistically significant. **(C).** Flow cytometric analysis of ERK1/2 phosphorylation in Jurkat T cells mixed with SEE-pulsed B cells purified from buffy coats of either healthy donors (HD B, n = 6) or peripheral blood of CLL patients with p66Shc expression above (high-p66, n = 4) or below (low-p66, n = 6) threshold, incubated for 15 min at 37°C and stained with anti-CD3 and anti-phospho-ERK1/2 antibodies. Unstimulated Jurkat T cells (ns) were used as control. One-way ANOVA test; **p ≤ 0.01; ***p ≤ 0.001; ****p ≤ 0.00001. **(D)**. Immunofluorescence analysis of NF-AT nuclear translocation in Jurkat T cells transiently transfected with a vector encoding GFP-NF-AT, then mixed with either healthy B cells (HD B, n = 6) or leukemic cells from CLL patients with p66Shc expression above (high-p66, n = 6) or below (low-p66, n = 6) threshold, incubated for 1 h at 37°C, and stained with anti-GFP antibodies. Unstimulated Jurkat T cell transfectants either unstimulated (ns) or stimulated for 30 min with 1 μg/mL A23187 (A23) were used as negative and positive controls, respectively. Data are expressed as % of cells with total GFP staining in the nuclei. Representative images (medial optical sections) are shown. Scale bar, 5 μm. MFI±SD. One-way ANOVA test; **p ≤ 0.01; ***p ≤ 0.001; ****p ≤ 0.00001.

CLL cells have a profound defect in the expression of the pro-apoptotic and pro-oxidant adaptor p66Shc (Capitani et al., 2010), that progressively further decreases during disease development (Capitani et al., 2019; Patrussi et al., 2021a). Since CLL cells suppress the ability of T cells to form ISs (Ramsay et al., 2012), we asked whether the p66Shc defect impinges on the IS-suppressive abilities of CLL cells. To this end, CLL cells were purified from peripheral blood of CLL patients and healthy donors and p66Shc mRNA was quantified by qRT-PCR (Supplementary Figure S1A). In agreement with previous results, the p66Shc mRNA levels were strongly decreased in CLL cells from our patient cohort compared to the levels detected in healthy B cells, with the lowest levels harbored by U-CLL cells (Supplementary Figure S1A, B). Based on their residual levels of p66Shc, CLL patients were then grouped in high p66Shc (high-p66) and low p66Shc (low-p66) expressing patients, according to an arbitrarily set threshold (0.25, corresponding to the median ddCt p66Shc mRNA of all CLL patients, Figure 1A). Cells from both high-p66 and low-p66 CLL patients, as well as B cells purified from healthy donors (HD), were pulsed with Staphylococcal enterotoxin E (SEE) and conjugated with Jurkat T cells. IS formation was impaired in conjugates of Jurkat T cells with CLL cells, as shown by the decreased frequency of conjugates displaying F-actin, and tyrosine phosphoprotein (p-Tyr) staining at the Jurkat T/CLL interface (Figure 1A). The frequency of conjugates displaying TCR/CD3 staining at the IS, which in Jurkat cells is mainly represented by a pool of TCR/CD3 complexes associated with recycling endosomes which moves beneath the synaptic membrane as a result of centrosome polarization (Soares et al., 2013; Onnis and Baldari, 2019) was also impaired (Figure 1A). Moreover, IS formation was significantly decreased in conjugates of Jurkat T cells with CLL cells with p66Shc expression below threshold compared to conjugates formed with CLL cells with p66Shc expression above threshold (Figure 1A). The frequency of F-actin^+^, TCR/CD3^+^ and p-Tyr^+^ conjugates significantly correlated with the residual p66Shc expression in CLL cells (Figure 1B), suggesting that the residual p66Shc expression in CLL cells affects their ability to form ISs with T cells. In agreement with their lowest p66Shc expression (Supplementary Figure S1B) (Capitani et al., 2010; Capitani et al., 2019; Patrussi et al., 2021a), U-CLL cells showed a significant impairment in the frequency of F-actin^+^, TCR/CD3^+^ and p-Tyr^+^ conjugates with Jurkat T cells when compared to M-CLL cells (Supplementary Figure S1C).

We then assessed whether the defective ability of CLL cells to form ISs with T cells also affects the activation state of downstream TCR signaling mediators. Phosphorylation of the mitogen-activated protein kinase ERK (phospho-ERK1/2) was impaired in Jurkat T cells conjugated with CLL cells with p66Shc expression above threshold when compared to Jurkat T cells conjugated with healthy B cells (Figure 1C), with a significant further decrease in conjugates of Jurkat T cells with CLL cells with p66Shc expression below threshold (Figure 1C). A similar impairment was observed when nuclear translocation of the transcription factor NF-AT was quantified by immunofluorescence in Jurkat T cells transiently transfected with a vector encoding the GFP-NF-AT fusion protein and then conjugated with either healthy B cells or CLL cells (Figure 1D). Our results demonstrate that the p66Shc-dependent IS defects observed in Jurkat T cells conjugated with CLL cells also affect downstream mediators of TCR signaling.

**FIGURE 2:**
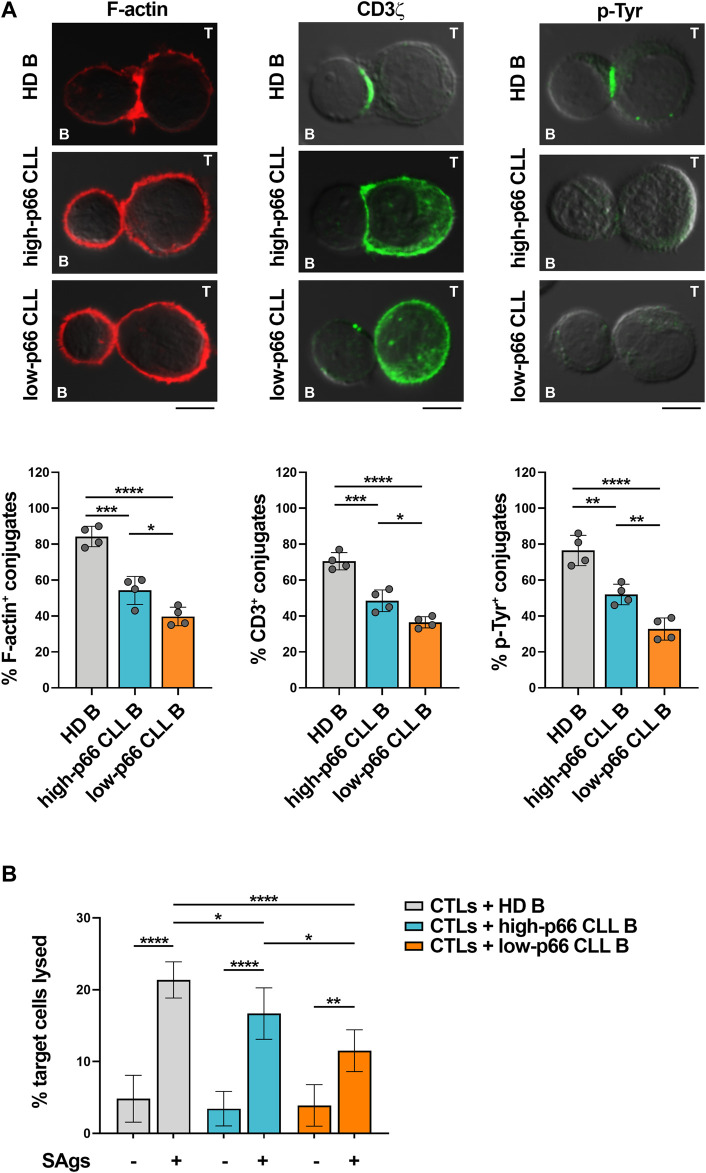
CLL cells with low p66Shc expression suppress IS formation and killing activities of CTLs. **(A)**. Immunofluorescence analysis of F-actin, CD3ζ and p-Tyr in CD8^+^ cells purified from buffy coats (n = 2), stimulated with anti-CD3/CD28 mAb-coated beads + IL-2 for 48 h and mixed with B cells purified from buffy coats of healthy donors (HD B, n = 4) or peripheral blood of CLL patients with p66Shc expression above (high-p66, n = 4) or below (low-p66 n = 4) threshold (0.25 median ddCt value, see Supplementary Figure S1A), pulsed with a combination of SEA, SEB, and SEE (SAgs) and incubated for 15 min at 37°C. Data are expressed as % of 15-min SAg-specific conjugates harboring staining at the IS (≥50 cells/sample). One-way ANOVA test; *p ≤ 0.05; **p ≤ 0.01; ***p ≤ 0.001; ****p ≤ 0.00001. Representative images (medial optical sections) of the CTL/B cell conjugates are shown. Scale bar, 5 μm. **(B)**. Flow cytometric analysis of target cell killing by CFSE-labelled CTLs, using as targets SAg-loaded B cells purified from buffy coats of healthy donors (HD B, n = 19) or peripheral blood of CLL patients with p66Shc expression above (high-p66, n = 5) or below (low-p66, n = 6) threshold, at a T:B cell ratio 10:1. Cells were cocultured for 4 h and stained with propidium iodide prior to processing for flow cytometry. Analyses were carried out gating on CFSE^−^/PI^+^ cells. The histogram shows the percentage (%) of target cells lysed. One-way ANOVA test; *p ≤ 0.05; **p ≤ 0.01; ****p ≤ 0.00001.

Defects in TCR signaling and IS formation result in impaired cytotoxic T cell (CTL) effector functions in CLL (Riches et al., 2013). We asked whether the p66Shc-dependent IS defects may result in impaired CTL-mediated killing. To address this point, CD8^+^ cells were immunopurified from buffy coats of healthy donors and differentiated to CTLs (Onnis et al., 2023). Similar to Jurkat T cells (Figure 1A), CTLs showed impaired ability to form ISs with CLL cells, with the highest impairment observed in conjugates formed with CLL cells with p66Shc expression below threshold (Figure 2A). Killing activity was then quantified in CFSE-stained CTLs incubated with SAg-pulsed leukemic or healthy B cells for 4 h, followed by flow cytometric analysis of propidium iodide-stained B cells. Consistent with the IS defects, CLL cells suppressed the ability of CTLs to kill target cells, with the highest suppressive activity harbored by CLL cells with p66Shc expression below threshold (Figure 2B).

Taken together, our data demonstrate that leukemic cells from patients with aggressive disease presentation and the lowest residual p66Shc expression suppress the ability of T lymphocytes to form ISs, to activate the TCR-dependent signaling pathway and to execute their effector functions.

### 3.2 The enhanced PD-L1 expression in CLL cells is caused at least in part by their p66Shc defect

**FIGURE 3:**
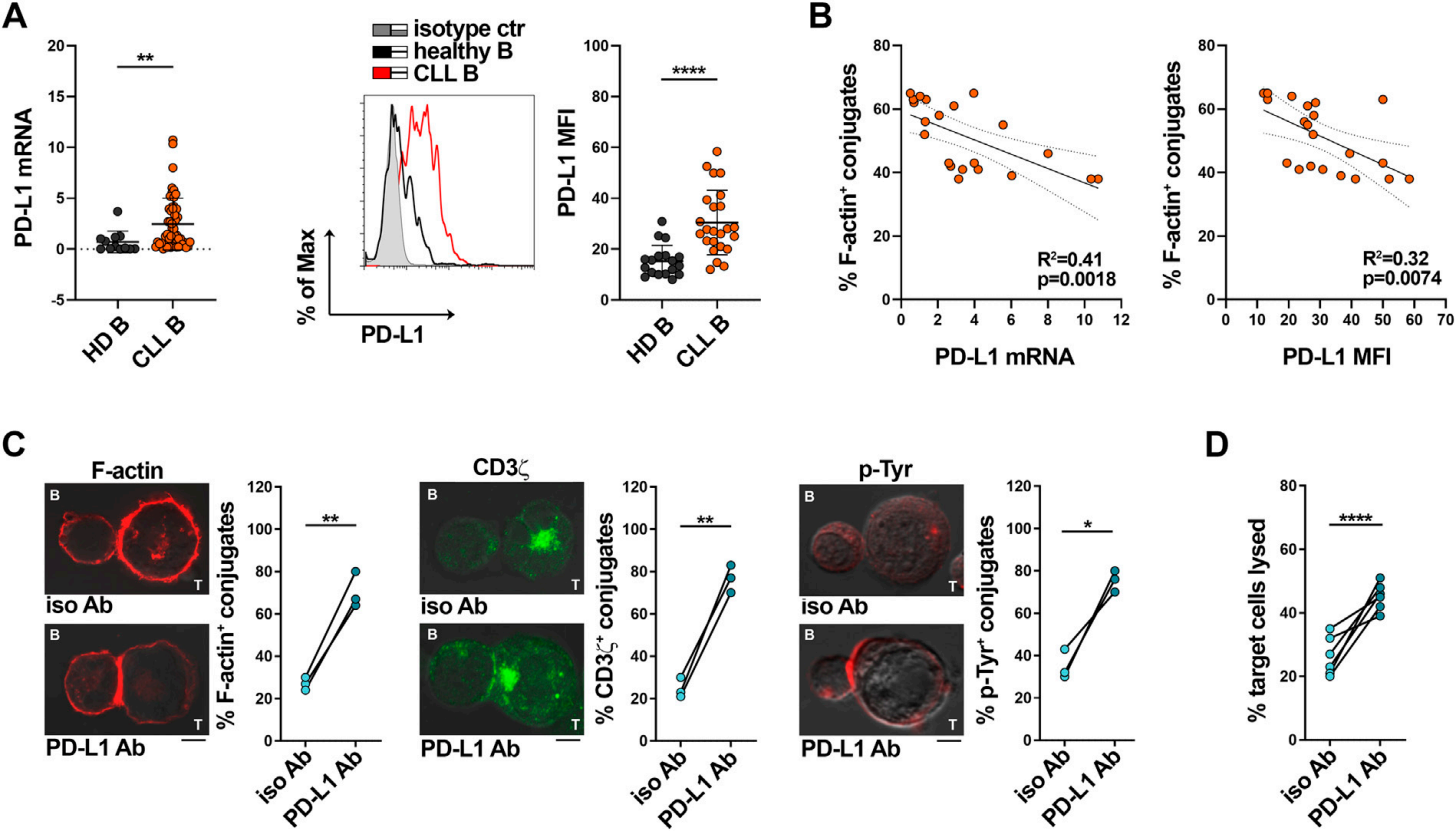
PD-L1 overexpression in CLL cells impairs IS formation with Jurkat T cells. **(A)** qRT-PCR analysis of mRNA (left) and flow cytometric analysis of surface (right) expression of PD-L1 in B cells purified from buffy coats of healthy donors (HD B, n = 12) or peripheral blood of CLL patients (CLL B, n = 52). A representative flow cytometric histogram is shown. Mann-Whitney Rank Sum test; **p ≤ 0.01; ****p ≤ 0.00001. **(B)**. Correlation between F-actin^+^ Jurkat/CLL B conjugates shown in Figure 1A and mRNA (left) and surface (right) levels of PD-L1 shown in **(A)** (n = 21). Simple linear regression; *p* ≤ 0.05 statistically significant. **(C)**. Immunofluorescence analysis of F-actin, CD3ζ and p-Tyr in Jurkat T cells mixed with B cells purified from peripheral blood of CLL patients, pulsed with SEE and incubated for 15 min at 37°C either in the absence or in the presence of anti-PD-L1 neutralizing antibodies. Data are expressed as % of 15-min SEE-specific conjugates harboring staining at the IS (≥50 cells/sample, n independent experiments = 3). Paired t-test; *p ≤ 0.05; **p ≤ 0.01. Representative images (medial optical sections) of the Jurkat T cell/B cell conjugates are shown. Scale bar, 5 μm. **(D)**. Flow cytometric analysis of target cell killing by CFSE-labelled healthy CTLs (n buffy coats = 3), using as targets B cells purified from peripheral blood of CLL patients (ratio T:B cells 10:1), pulsed with SAgs either in the absence or in the presence of anti-PD-L1 neutralizing antibodies. Cells were cocultured for 4 h and stained with propidium iodide prior to processing for flow cytometry. Analyses were carried out gating on CFSE^−^/PI^+^ cells. The histogram shows the percentage (%) of target cells lysed (n CLL patients = 6). One-way ANOVA test; ****p ≤ 0.00001.

Multiple inhibitory ligands that are upregulated in CLL cells have been implicated in the suppression of IS formation and T cell effector functions, among which the PD-1 ligand PD-L1 (Ramsay et al., 2008; Riches et al., 2013). Accordingly, leukemic cells isolated from our CLL patient cohort express enhanced mRNA and surface PD-L1 compared to healthy B cells (Figure 3A). Moreover, both mRNA and surface expression of PD-L1 inversely correlated with the ability of leukemic B cells to form ISs with Jurkat T cells (Figure 3B, Supplementary Figure S2). To confirm the inverse relationship between PD-L1 expression in leukemic cells and IS formation, SEE-pulsed CLL cells were conjugated with Jurkat T cells in the presence of anti-PD-L1 neutralizing antibodies. PD-L1 neutralization recovered IS formation, as shown by the enhanced frequency of F-actin^+^, CD3ζ^+^ and p-Tyr^+^ conjugates (Figure 3C). Moreover, PD-L1 blockade recovered the killing activity of CTLs toward SAg-pulsed CLL cells (Figure 3D), further supporting the notion that the suppressive activity of CLL cells relies at least in part on their PD-L1 expression levels.

**FIGURE 4:**
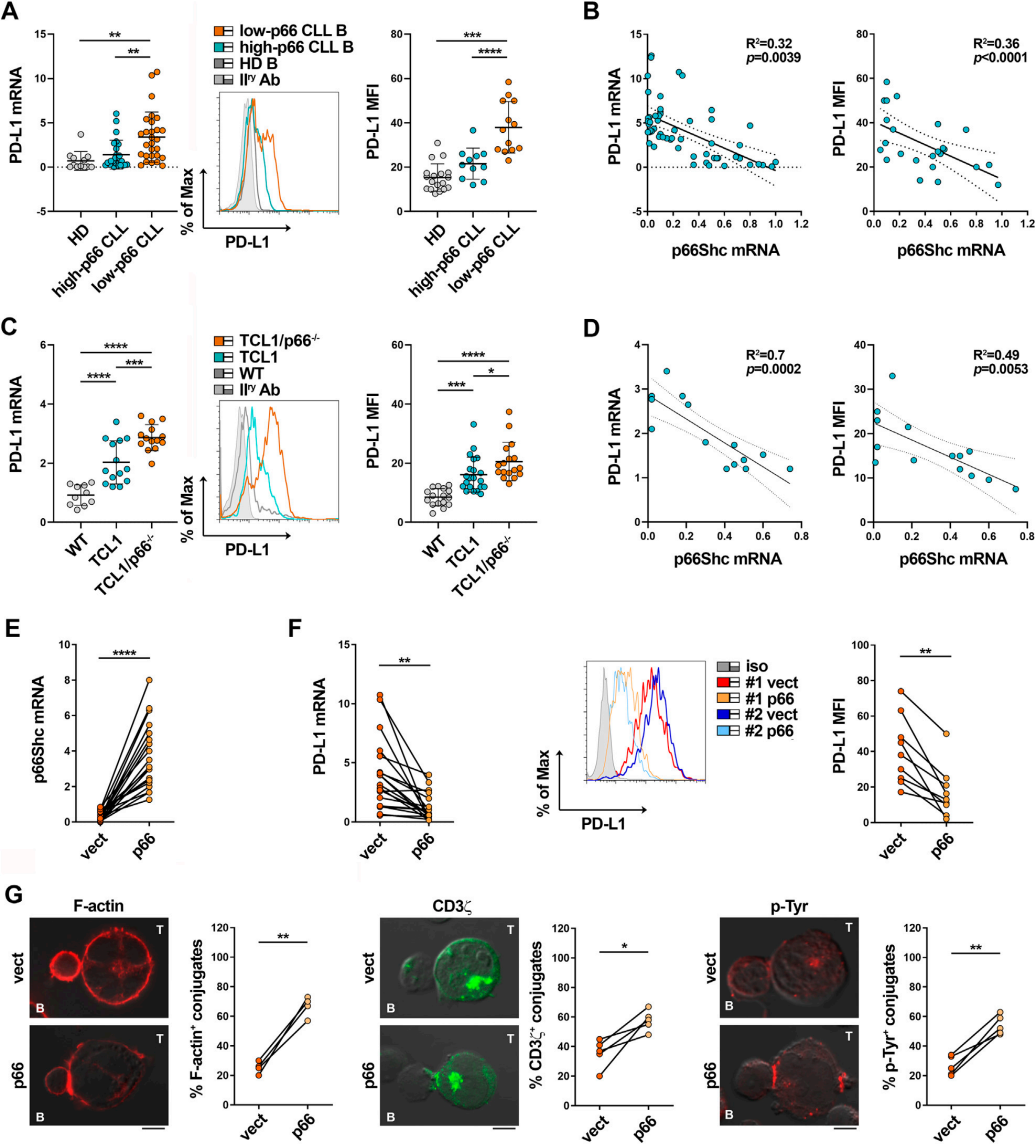
The p66Shc defect accounts for PD-L1 overexpression in CLL cells. **(A)** qRT-PCR analysis of mRNA (left) and flow cytometric analysis of surface (right) expression of PD-L1 in B cells purified from buffy coats of healthy donors (HD B, n = 12) or peripheral blood of CLL patients with p66Shc expression above (high-p66, n = 26) or below (low-p66, n = 26) threshold (0.25 median ddCt value, see Supplementary Figure S1A). A representative flow cytometric histogram is shown. One-way ANOVA test; **p ≤ 0.01; ***p ≤ 0.001; ****p ≤ 0.00001. **(B)**. Correlation between mRNA (left) and surface (right) levels of PD-L1 shown in **(A)** and mRNA levels of p66Shc in CLL cells (n = 21). Simple linear regression; *p* ≤ 0.05 statistically significant. **(C)**. qRT-PCR analysis of mRNA (letf) and flow cytometric analysis of surface (right) expression of PD-L1 in B cells isolated from spleens of either wild-type (n = 10), Eμ-TCL1 (n = 14) or Eμ-TCL1/p66Shc^−/−^ (n = 13) mice. Representative flow cytometric histograms are shown (Mann Whitney Rank Sum test). *p ≤ 0.05; ***p ≤ 0.001; ****p ≤ 0.0001. **(D)**. Correlation between mRNA (left) and surface (right) levels of PD-L1 shown in **C** and mRNA levels of p66Shc in B cells isolated from Eμ-TCL1 mice (n = 14). Simple linear regression; *p* ≤ 0.05 statistically significant. **(E)**. qRT-PCR analysis of p66Shc mRNA in B cells purified from CLL patients and transfected with either empty (vect) or p66Shc-encoding (p66) vectors (n = 19; paired t-test, ****p ≤ 0.0001). **(F)**. qRT-PCR analysis of mRNA (left) and flow cytometric analysis of surface (right) expression of PD-L1 in B cells purified from CLL patients and transfected with either empty (vect) or p66Shc-encoding (p66) vectors (n ≥ 9; paired t-test, **; *p* ≤ 0.01). **(G)**. Immunofluorescence analysis of F-actin, CD3ζ and p-Tyr in Jurkat T cells mixed with B cells purified from peripheral blood of CLL patients and transfected with either empty (vect) or p66Shc-encoding (p66) vectors, pulsed with SEE and incubated for 15 min at 37°C. Data are expressed as % of 15-min SEE-specific conjugates harboring staining at the IS (≥50 cells/sample, n = 4). Paired t-test; *p ≤ 0.05; **p ≤ 0.01. Representative images (medial optical sections) of the Jurkat T cell/B cell conjugates are shown. Scale bar, 5 μm.

P66Shc deficiency in CLL cells impinges on the expression of a number of CLL-critical genes, including genes encoding lymphoid homing receptors, and cytokines such as IL-9 (Capitani et al., 2012; Patrussi et al., 2015; Patrussi et al., 2021a; Tatangelo et al., 2022). We asked whether it also promotes PD-L1 expression in CLL cells. Both mRNA and surface PD-L1 were significantly enhanced in low-p66Shc compared to high-p66Shc CLL cells (Figure 4A). Moreover, PD-L1 expression inversely correlated with the residual p66Shc expression in CLL cells (Figure 3B). In agreement with their lowest p66Shc expression (Supplementary Figure S1B) (Capitani et al., 2010; Capitani et al., 2019; Patrussi et al., 2021a), U-CLL cells showed a significant enhancement in PD-L1 expression when compared to M-CLL cells (Supplementary Figure S3A).

These results were recapitulated in the Eμ-TCL1 mouse model of CLL (Bichi et al., 2002), where leukemic cells that accumulate in aged mice show decreased expression of p66Shc and prolonged survival (Patrussi et al., 2019). Both mRNA and surface PD-L1 were enhanced in leukemic cells isolated from spleens of Eμ-TCL1 mice compared to wild-type mice (Figure 4C), especially those with the worst disease presentation (>40% CD5^+^CD19^+^IgM^+^ cells in peripheral blood (Patrussi et al., 2019)) (Supplementary Figure S3B), and inversely correlated to p66Shc mRNA expression (Figure 4D). The tight correlation between p66Shc and PD-L1 expression in Eμ-TCL1 mice is corroborated by the further increase in the amounts of PD-L1 displayed by leukemic cells isolated from spleens of Eμ-TCL1/p66^−/−^ mice (Figures 4C,D). Hence, the p66Shc defect accounts at least in part for the enhanced PD-L1 expression in CLL cells.

We asked whether p66Shc reconstitution in CLL cells could normalize PD-L1 expression and IS formation. Leukemic cells were isolated from CLL patients and transiently nucleofected with vectors encoding p66Shc (p66). Cells transfected with empty vector were used as control (vect) (Figure 4E). Reconstitution of CLL cells with wild-type p66Shc decreased PD-L1 expression compared to the empty vector control (Figures 4E,F). Notably, p66Shc reconstitution did not affect the expression of the inhibitory ligands CD200, CD276 and CD270 (Supplementary Figure S4) which, similar to PD-L1, are upregulated in CLL cells and contribute to the IS defects observed in CLL (Ramsay et al., 2012), ruling them out as mediators of the p66Shc-dependent IS dysfunctions. Transfectants were then pulsed with SEE and conjugated with Jurkat T cells. Reconstitution of CLL cells with p66Shc significantly enhanced IS formation, as shown by the increased frequency of conjugates displaying F-actin, TCR/CD3 and p-Tyr staining at the Jurkat T/CLL interface (Figure 4G). Altogether, our results support the notion that the p66Shc defect in CLL cells contributes to their IS-suppressive activities by modulating PD-L1 expression.

### 3.3 The pro-oxidant activity of p66Shc controls PD-L1 expression in CLL cells

**FIGURE 5:**
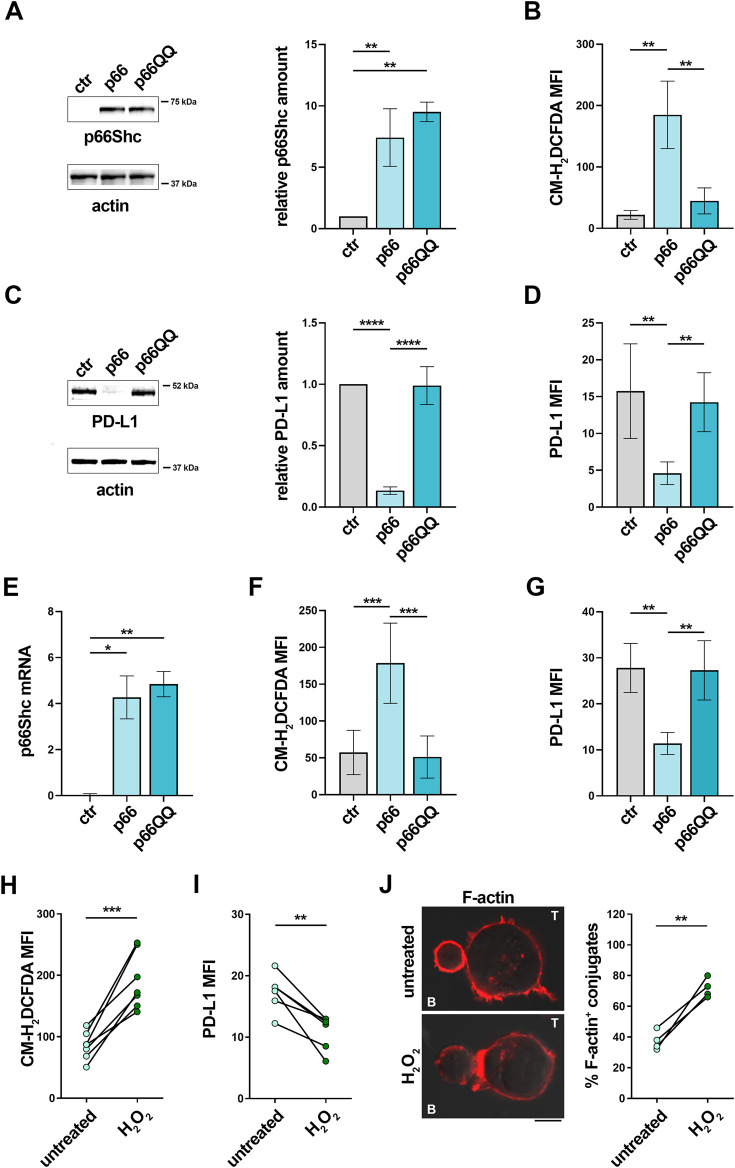
The p66Shc deficiency-related impairment in homeostatic ROS contributes to the enhanced PD-L1 expression in CLL cells. **(A)**. Immunoblot analysis with anti-Shc antibodies of postnuclear supernatants of MEC-1 cells stably transfected with empty vector (ctr) or with a vector encoding either wild-type (p66) or mutated (p66QQ) p66Shc. The stripped filters were reprobed with anti-actin antibodies. Molecular weights (kDa) are indicated on the right of the panel. The quantification of three independent experiments is shown on the right. **(B)**. Flow cytometric analysis of ROS intracellular content in MEC-1 transfectants stained with CM-H_2_DCFDA (n independent experiments ≥5). **(C)**. Immunoblot analysis with anti-PD-L1 antibodies of postnuclear supernatants of MEC-1 transfectants. The stripped filters were reprobed with anti-actin antibodies. Molecular weight markers (kDa) are indicated on the right of the panel. The quantification of three independent experiments is shown on the right. **(D)**. Flow cytometric analysis of surface expression of PD-L1 in MEC-1 transfectants (n ≥ 5). **(E)**. qRT-PCR analysis of mRNA expression of p66Shc in B cells purified from CLL patients and transfected with empty vector (ctr) or with a vector encoding either wild-type (p66) or mutated (p66QQ) p66Shc (n = 4). **F, (G)**. Flow cytometric analysis of ROS intracellular content **(F)** and surface expression of PD-L1 **(G)** in CLL cell transfectants **(F)**: n = 6; **(G)**; n = 4). **(A-G)**: One-way ANOVA test; *p ≤ 0.05; **p ≤ 0.01; ***p ≤ 0.001; ****p ≤ 0.00001. **(H, I)**. Flow cytometric analysis of ROS intracellular content **(H)** and surface expression of PD-L1 **(I)** in B cells purified from peripheral blood of CLL patients, either untreated or treated for 6 h **(H)**; n = 6) or 24 h **(I)**; n = 6) with 100 μM H_2_O_2_. **(J)**. Immunofluorescence analysis of F-actin in Jurkat T cells mixed with B cells purified from peripheral blood of CLL patients and treated for 24 h with 100 μM H_2_O_2_, pulsed with SEE and incubated for 15 min at 37°C. Data are expressed as % of 15-min SEE-specific conjugates harboring staining at the IS (≥50 cells/sample, n = 4). **(H-J)**: Paired t-test; *p ≤ 0.05; **p ≤ 0.01; ***p ≤ 0.001. Representative images (medial optical sections) of the Jurkat T cell/B cell conjugates are shown. Scale bar, 5 μm.

We have recently reported that p66Shc modulates gene expression through its ROS-elevating activity (Tatangelo et al., 2022). To address the potential role of the pro-oxidant activity of p66Shc in PD-L1 expression, we used MEC-1 cells, which do not express p66Shc due to promoter methylation (Cattaneo et al., 2016), stably transfected with either empty vector (ctr), or wild-type p66Shc (p66), or a ROS-defective p66Shc mutant carrying E to Q substitutions at positions 132–133 (p66QQ) (Tatangelo et al., 2022) (Figure 5A). Flow cytometric analysis of homeostatic ROS production in the CM-H_2_DCFDA-loaded MEC-1 transfectants confirmed an enhanced ROS production in p66Shc-expressing cells, but not in cells expressing p66ShcQQ, compared to control cells (Figure 5B). PD-L1 expression was strongly decreased in the MEC transfectant expressing wild-type p66Shc compared to the empty vector control, as assessed by immunoblot and flow cytometry (Figures 5C,D). Interestingly, at variance with wild-type p66Shc, expression of the ROS-defective p66Shc mutant did not affect PD-L1 expression (Figures 5C,D), suggesting that p66Shc regulates PD-L1 expression through its ROS-elevating activity.

By elevating intracellular ROS, p66Shc suppresses the transcriptional activity of NF-κB (Tatangelo et al., 2022). Since PD-L1 expression is regulated by NF-κB (Lim et al., 2016; Antonangeli et al., 2020), we asked whether the p66Shc- and ROS-dependent PD-L1 reduction is mediated by NF-κB. To address this point, PD-L1 expression was quantified by flow cytometry in MEC-1 transfectants treated for 24 h with 1 μM IT-901, a NF-κB inhibitor that has been proven efficient in inhibiting the transcriptional activity of NF-κB in CLL cells (Vaisitti et al., 2017). In agreement with previous results (Tatangelo et al., 2022), NF-κB activation, assessed by flow cytometric analysis of transfectants using anti-phospho-NF-κB p65 (Ser536) antibodies, was significantly impaired in the p66 transfectant, (Supplementary Figure S5A). IT-901 reduced NF-κB phosphorylation (SSupplementary Figure S5A) in the control transfectant to levels similar to p66Shc-expressing cells (Supplementary Figure S1A, B). Interestingly, NF-κB inhibition in control cells strongly decreased PD-L1 expression (Supplementary Figure S1B). The ROS-defective p66Shc mutant p66QQ did not affect either NF-κB phosphorylation or PD-L1 expression (Supplementary Figure S5A, B). Hence, NF-κB inhibition elicited by reconstitution of wild-type p66Shc, but not the ROS-defective p66QQ mutant, suppresses PD-L1 expression.

To validate these findings in CLL cells, where low intracellular ROS are causally associated to their p66Shc expression defect (Patrussi et al., 2019; Tatangelo et al., 2022), leukemic cells isolated from CLL patients were transiently nucleofected with vectors encoding either wild-type p66Shc (p66) or the ROS defective p66ShcQQ mutant (p66QQ) (Figure 5E) and PD-L1 expression was assessed by flow cytometry 24 h after transfection. In agreement with our previous results (Patrussi et al., 2019), reconstitution of CLL cells with wild-type p66Shc, but not with the p66ShcQQ mutant, enhanced intracellular ROS compared to the empty vector control (Figure 5F). Under these conditions, PD-L1 expression was selectively downregulated in the presence of wild-type p66Shc (Figure 5G).

Interestingly, the NF-κB inhibitor IT-901 decreased PD-L1 expression in CLL cells transfected with either empty vector or vector encoding the ROS-defective p66ShcQQ mutant to levels similar to CLL cells transfected with wild-type p66Shc (Supplementary Figure S5C, D), supporting the notion that, by restoring ROS production, forced p66Shc expression in CLL cells hampers NF-κB activation, thereby lowering their abnormal PD-L1 expression.

Our data suggest that PD-L1 overexpression in CLL cells can be counteracted by enhancing intracellular ROS levels. To further corroborate this notion, we measured PD-L1 in CLL cells treated for 24 h with 100 μM H_2_O_2_ as exogenous source of ROS. This treatment reduced PD-L1 expression (Figures 5H,I) and recovered their ability to form ISs with Jurkat T cells (Figure 5L). Taken together, these results demonstrate that the p66Shc-dependent decrease in intracellular ROS in CLL cells enhances NF-κB transcriptional activity, which in turn contributes to promote PD-L1 expression, thereby suppressing their ability to form ISs with T lymphocytes.

## 4 Discussion

The incomplete knowledge concerning the molecular circuitries that fuel disease progression in the tumoral microenvironment makes the management of CLL challenging (Arruga et al., 2020). Both direct and indirect mechanisms implemented in the lymphoid niche converge to promote leukemic cell survival and resistance to therapy (Burger and Gribben, 2014; Allegra et al., 2020; Patrussi et al., 2021b; Capitani et al., 2021). Some of them are focused on disabling the anti-tumoral activities of T lymphocytes by hampering their ability to form ISs with leukemic cells and to selectively kill them in an antigen-dependent manner (Ramsay et al., 2008; Burger and Gribben, 2014; Capitani et al., 2021). In the tumor microenvironment of CLL both the conversion of functional to exhausted T cells overexpressing the exhaustion marker PD-1 (Riches et al., 2013), and PD-L1 overexpression on the surface of leukemic cells, concur to exacerbate the physiological PD-1/PD-L1 inhibitory axis and hamper TCR-mediated T cell activation (Riches et al., 2013). The molecular mechanisms underlying PD-L1 overexpression in CLL cells remain to be clarified. Here we demonstrate that the profound defect in the expression of the pro-apoptotic and pro-oxidant adaptor p66Shc in CLL cells (Capitani et al., 2010) accounts at least in part for their enhanced expression of PD-L1. The p66Shc-dependent PD-L1 increase in CLL cells in turn prevents T lymphocytes from forming ISs with leukemic cells themselves, thereby facilitating the evasion of leukemic cells from T cell-mediated surveillance.

Notwithstanding the fact that PD-L1 expression correlates with disease stage and prognosis in some tumoral contexts, among which lung adenocarcinoma, nasopharyngeal carcinoma and non-small cell lung cancer (Nie et al., 2021; Zhou et al., 2023), the relationship between surface PD-L1 and disease parameters remains largely unknown. Here we demonstrated that leukemic cells from U-CLL patients, that develop aggressive forms of the disease (Kipps et al., 2017), as well as from Eμ-TCL1 mice with overt leukemia, express the highest surface amounts of PD-L1 and harbor the most relevant IS-suppressive activity. These results provide new evidence of the relationship between disease stage and PD-L1 expression in CLL, and suggest that p66Shc deficiency in CLL cells from patients with aggressive disease impinges in T cell suppression by enhancing PD-L1 expression.

Genetic aberrations, such as ionizing radiation-induced double strand breaks, have been implicated in PD-L1 overexpression in cancer cells (Zhou et al., 2023). Moreover, pro-inflammatory stimuli such as interferons, as well as altered oncogenic signaling pathways, among which the Ras/mitogen activated protein kinase (MAPK) and the Phosphatidyl inositol-3-kinase (PI3K)/Akt pathways, also promote PD-L1 overexpression (Zhou et al., 2023). Interestingly, a complex interplay between intracellular ROS and PD-L1 expression in cancer cells has recently come to light (Bailly, 2020). Depending on their targets, ROS either up- or downregulate PD-L1 expression in cancer cells. ROS-elevating hypoxic conditions, that promote both expression, activation and stabilization of the transcription factors HIF-1α, NF-κB and Yes-associated protein 1 (Yap1) (Kang et al., 2020), are frequently associated to elevated membrane expression of PD-L1 on cancer cells of solid tumors such as pulmonary pleomorphic carcinoma (Chang et al., 2016), endometrial cancer (Tawadros and Khalafalla, 2018) and non-small-cell lung cancer (Guo et al., 2019). However, pro-oxidant drugs such as the biguanide compounds metformin and phenformin, that induce oxidative stress-mediated cancer cell apoptosis (Zhao et al., 2019), attenuate PD-L1 expression via the Hippo signaling pathway in colorectal cancer and melanoma (Zhang et al., 2019). In B lymphocytes, p66Shc has a frank ROS-elevating activity which modulates the expression of genes encoding trafficking receptors such as CCR7 and S1PR1 (Capitani et al., 2012; Patrussi et al., 2019; Patrussi et al., 2020). We have recently shown that the p66Shc expression defect in CLL cells leads to a decrease in the intracellular ROS levels, which in turn abnormally enhance the transcriptional activity of the ROS-sensitive p65 subunit of NF-κB (Tatangelo et al., 2022) and promotes IL-9 release by CLL cells (Patrussi et al., 2021a), thereby contributing to shape the tumor niche to a pro-survival one (Patrussi et al., 2021a; Patrussi et al., 2021b). Here we demonstrate that the p66Shc-dependent ROS defect also controls PD-L1 overexpression in CLL cells. We moreover provide evidence that the drop in intracellular ROS levels of CLL cells indirectly impinges on leukemic cell survival not only by promoting their recruitment to and entrapment in the pro-survival stromal niche (Capitani et al., 2012; Patrussi et al., 2021a; Tatangelo et al., 2022), but also by preventing T cells from forming ISs with leukemic cells, thereby suppressing their anti-tumor activities. Hence, therapeutical approaches able to restore intracellular ROS might be explored to overcome PD-L1 overexpression and T cell dysfunctions in CLL.

It is however noteworthy that ROS are highly reactive compounds whose intracellular concentration must be tightly controlled to avoid protein, DNA, and lipid damage. (Yu et al., 2020). Direct pharmacological targeting of cell-intrinsic ROS-modulating enzymes in CLL cells might therefore be hardly feasible due to uncontrolled side effects (Wang et al., 2021). Here we demonstrate that reconstitution of p66Shc, whose expression is profoundly impaired in CLL cells (Capitani et al., 2010), but not of the ROS-defective p66ShcQQ mutant, leads to an elevation in intracellular ROS and normalizes PD-L1 expression, restoring the ability of T cells to form ISs with leukemic cells by removing one of the most important TCR signaling blocks.

Immunotherapy with Chimeric Antigen Receptor (CAR)-engineered T cells has emerged as a promising treatment option for high-risk relapsed/refractory CLL patients (Cassioli et al., 2022). However, as opposed to other B cell malignancies such as B-ALL (Maude et al., 2018) and large B cell lymphoma (Locke et al., 2019), where it has already been approved for clinical application, the beneficial use of CAR T cells in CLL is still debated and the number of CAR T cell-treated CLL patients who reached complete remission in the context of clinical trials is still limited (Cassioli et al., 2022). Although the reason for this therapeutic failure remains to be elucidated, the molecular mechanisms implemented by tumoral cells to overexpress inhibitory ligands such as PD-L1 and to suppress IS formation and anti-tumoral activities of T cells may contribute to also suppress CAR T cells. An in-depth understanding of the mechanisms exploited by CLL cells to enhance their IS-suppressive potential and turn off T cell (and CAR T cell) anti-tumor activity is therefore warranted to expand our treatment options.

## Data Availability

The original contributions presented in the study are included in the article/Supplementary Material, further inquiries can be directed to the corresponding author.
