# Supplementary material for: p66Shc deficiency in CLL cells enhances PD-L1 expression and suppresses immune synapse formation
Source: Front Cell Dev Biol. 2024 Jan 23;12:1297116. doi: 10.3389/fcell.2024.1297116 (PMC10883382; doi:10.3389/fcell.2024.1297116)
Supplement: Supplementary file 1 [file DataSheet1.docx]

Supplementary Material

# 1. Supplementary Figures


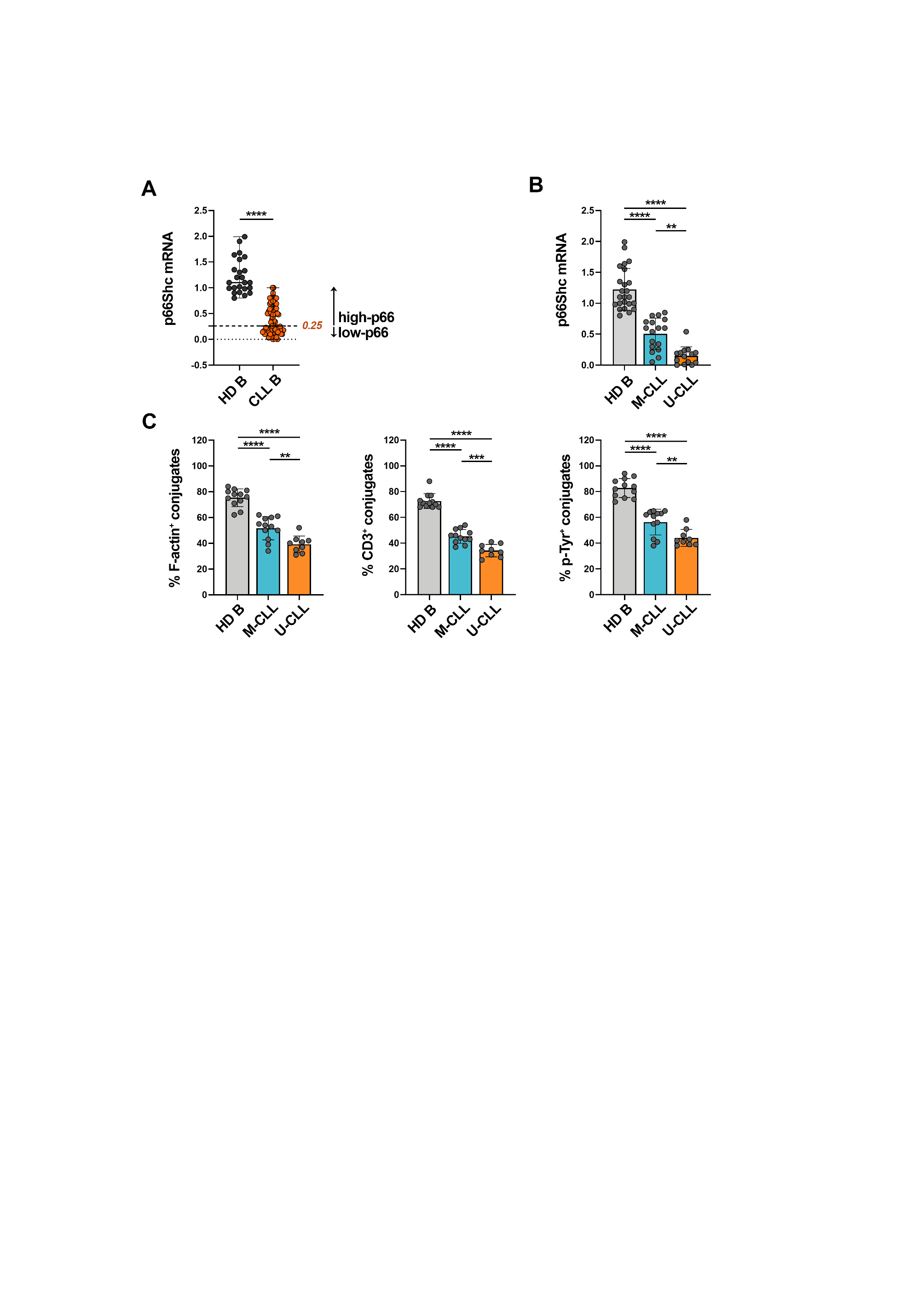


**Supplementary Figure 1.**

(**A**)**.** Quantitative RT-PCR analysis of p66Shc mRNA in B lymphocytes purified from peripheral blood of healthy donors (HD B; n=24) and CLL patients (CLL B; n=45). 0.25, the median ddCt of p66Shc mRNA in CLL samples, was used as threshold to identify high-p66 and low-p66 CLL cells. Mann Whitney Rank Sum test; *p*≤0.0001, ****. (**B**). p66Shc mRNA in B lymphocytes purified from peripheral blood of healthy donors and CLL patients shown in (**A**), grouped in M-CLL (n=17) and U-CLL (n=14) patients according to their mutational *IGHV* status. (**C**). Immunofluorescence analysis of F-actin, CD3ζ and p-Tyr in Jurkat T cells mixed with B cells purified from peripheral blood of healthy donors (HD B, n=12) or CLL patients grouped in M-CLL (n=12) and U-CLL (n=9) patients according to their *IGHV* mutational status, pulsed with SEE and incubated with for 15 min at 37°C. Data are expressed as % of 15-min SEE-specific conjugates harboring staining at the IS (≥ 50 cells/sample). One-way ANOVA test with Multiple Comparison; *p*≤0.01, **. *p*≤0.0001, ****.


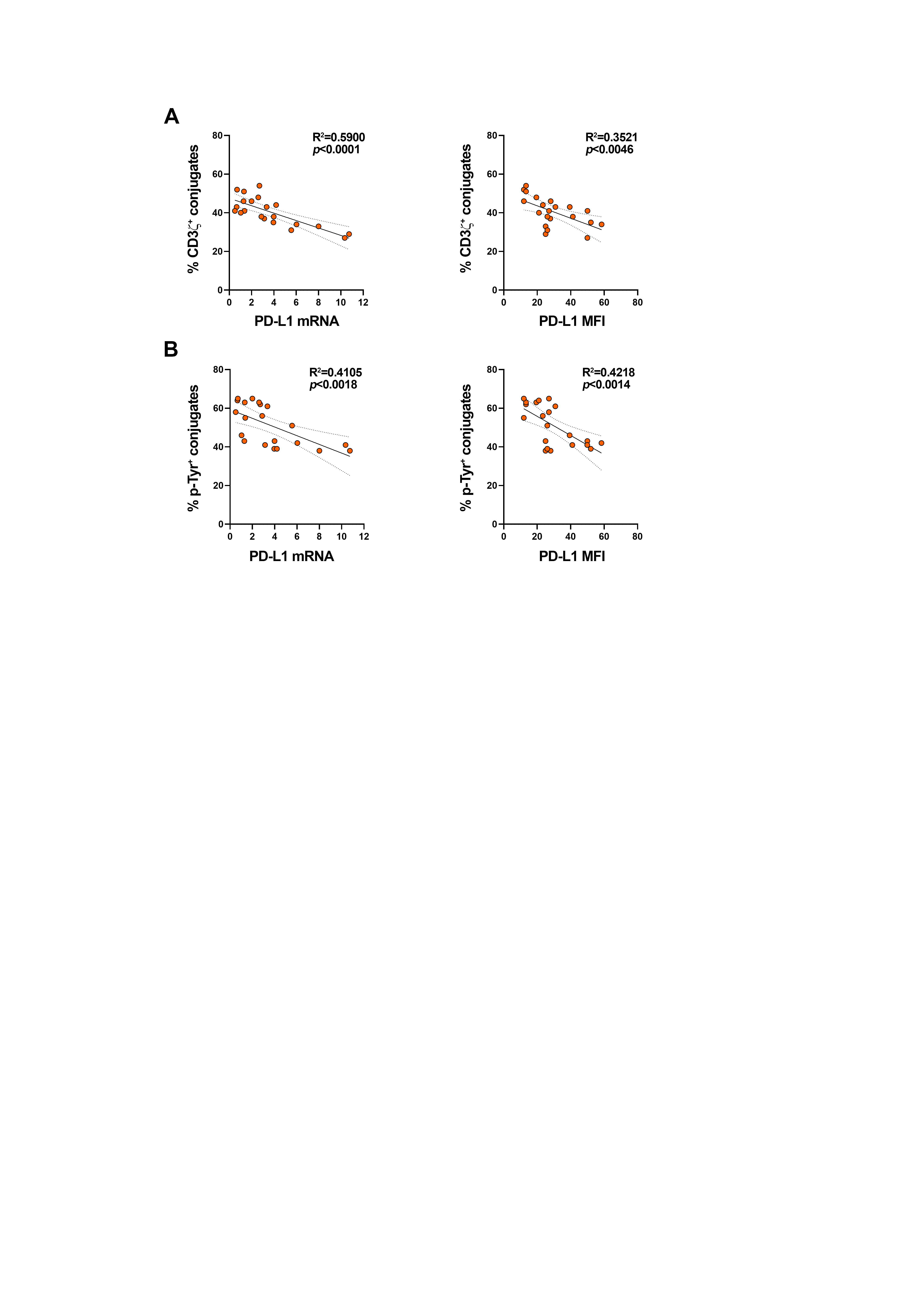


**Supplementary Figure 2.**

(**A, B**)**.** Correlation between CD3ζ^+^ (**A**) and p-Tyr^+^ (**B**) Jurkat/CLL B conjugates shown in Figure 1A and mRNA (left) and surface (right) levels of PD-L1 shown in Figure 2A (n=21). Simple linear regression; *p*≤0.05 statistically significant.

**
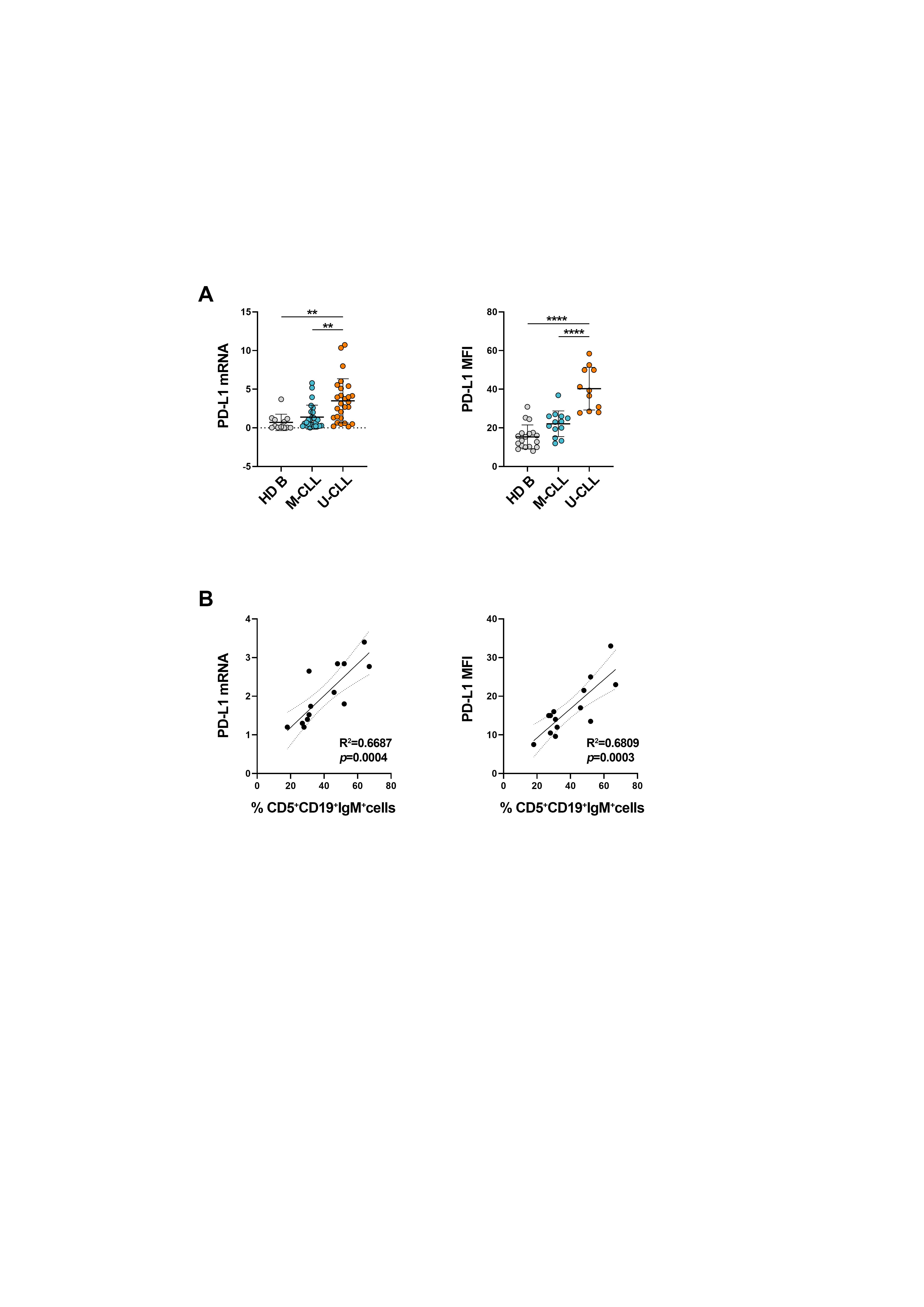
**

**Supplementary Figure 3. A.** qRT-PCR analysis of mRNA (left) and flow cytometric analysis of surface (right) expression of PD-L1 in B lymphocytes purified from peripheral blood of healthy donors (HD B) and CLL patients grouped in M-CLL and U-CLL patients according to their *IGHV* mutational status. One-way ANOVA test; ** *p*≤0.01; **** *p*≤0.00001. **B**. Correlation between the percentage of CD5^+^CD19^+^IgM^+^ cells in peripheral blood of Eμ-TCL1 mice and mRNA (left) and surface (right) levels of PD-L1 in leukemic cells isolated from spleens of the respective mice. Simple linear regression; *p*≤0.05 statistically significant.

**
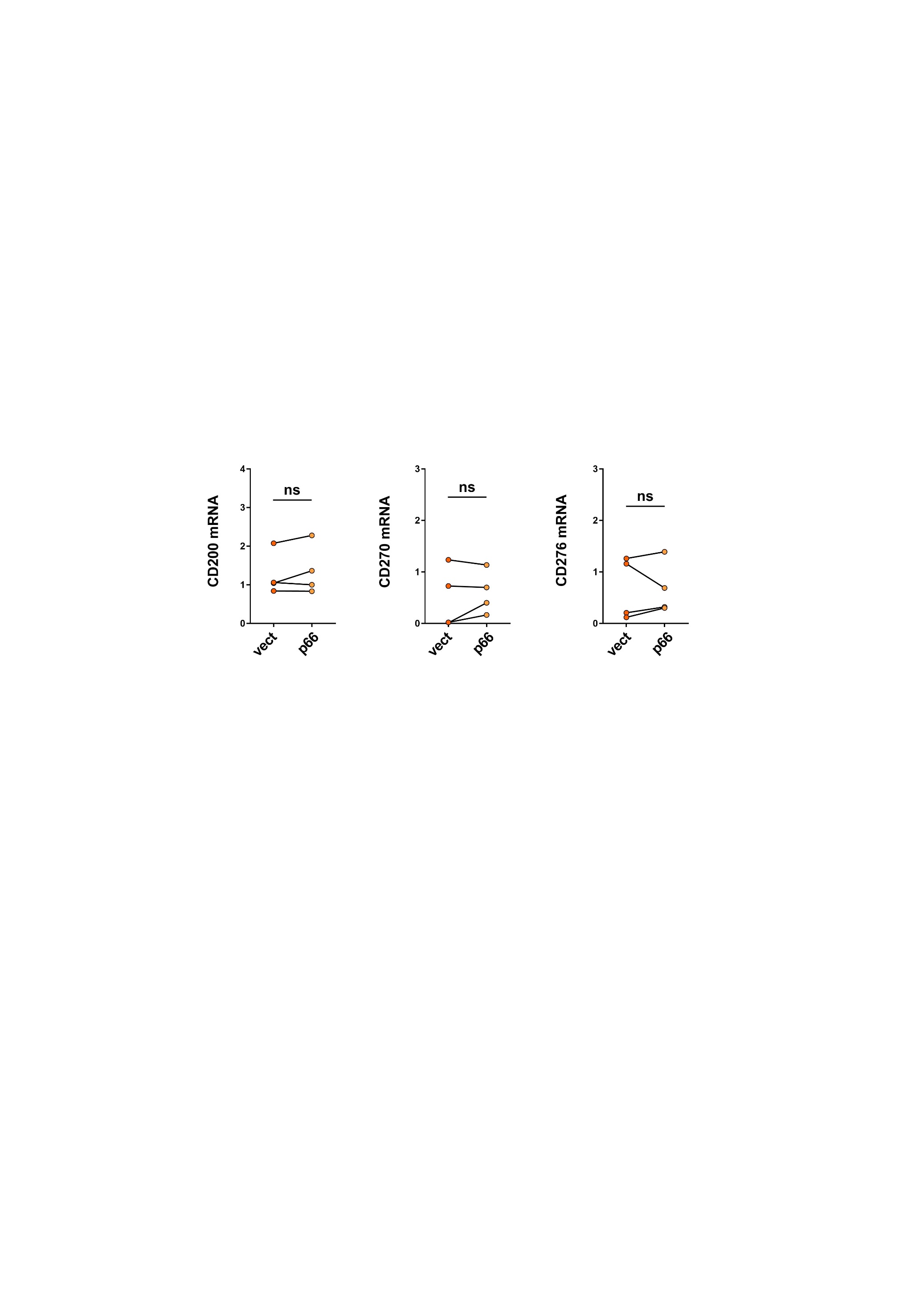
**

**Supplementary Figure 4.** qRT-PCR analysis of CD200, CD270 and CD276 mRNA in B cells purified from CLL patients and transfected with either empty (vect) or p66Shc-encoding (p66) vectors. (n=4; paired t test).

**
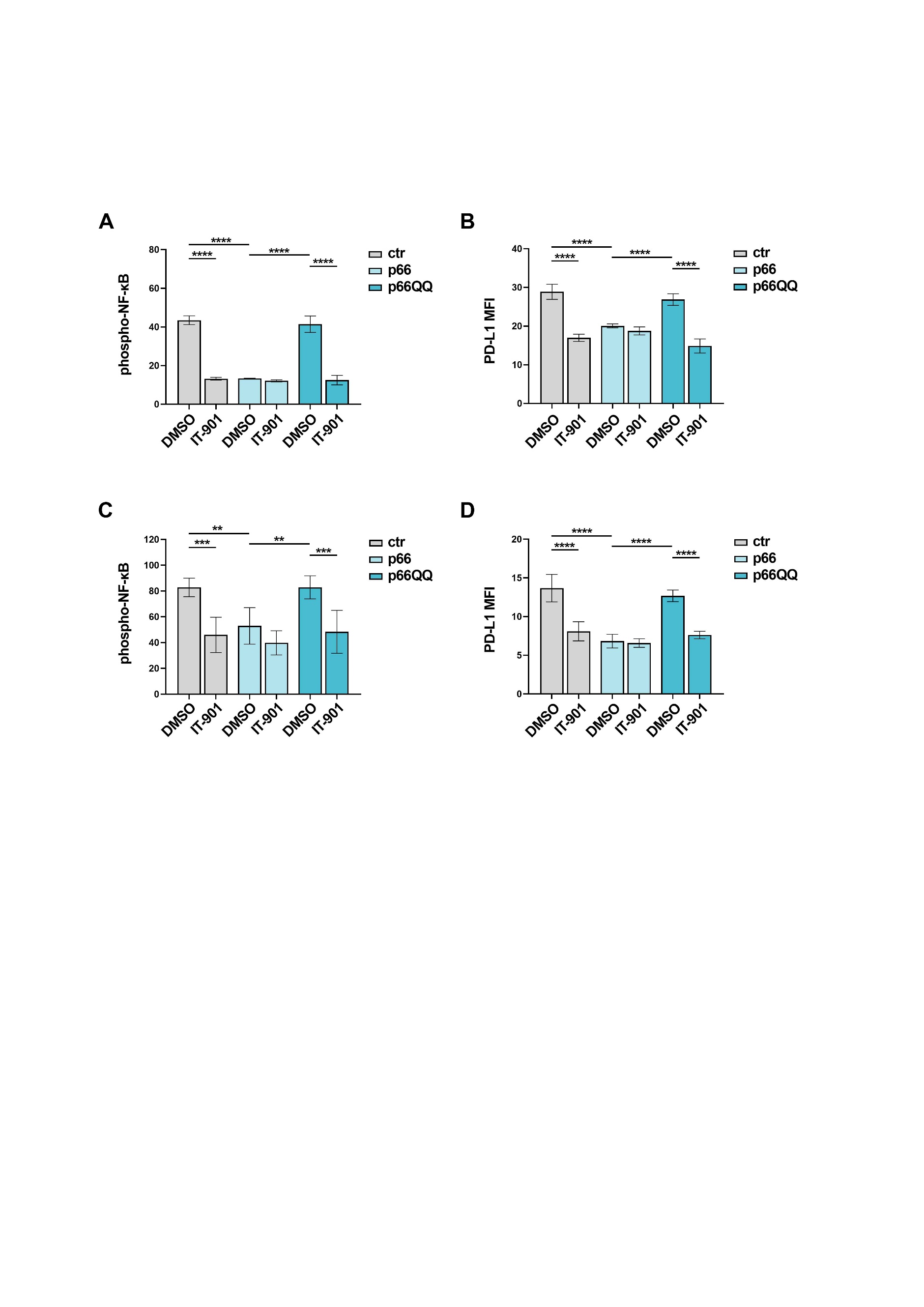
**

**Supplementary Figure 5. A, B.** Flow cytometric analysis of phospho-NF-κB (Ser536) (**A**) and PD-L1 expression (**B**) in MEC-1 cells stably transfected with empty vector (ctr) or with a vector encoding either wild-type (p66) or mutated (p66QQ) p66Shc, treated for 30 min (**A**) or 24 h (**B**) with either DMSO or 1 µM IT-901 (n independent experiments=4). **C, D.** Flow cytometric analysis of phospho-NF-κB (Ser536) (**C**) and PD-L1 expression (**D**) in B lymphocytes purified from peripheral blood of CLL patients (CLL), transiently transfected with the empty vector (vect) or a vector encoding either wild-type (p66) or mutated (p66QQ) p66Shc, treated for 30 min (**C**) or 24 h (**D**) with either DMSO or 1 µM IT-901 (n CLL patients=4). Mean±SD. Two-way ANOVA test, Multiple Comparison. ****; p≤0.001, ***; p≤0.01, **; p≤0.05, *.

**2. Supplementary Tables**

**Supplementary Table 1. List of reagents and antibodies used in this study.**

| Reagent | Host | Clone | Source | Cat. No. | Concentration |
| --- | --- | --- | --- | --- | --- |
| Fc-Block | rat | 2.4G2 | BD Biosciences | 553141 | 1:50 |
| PerCP-Cy5.5 IgM antibody | rat | RMM-1 | BioLegend | 406512 | 1:30 |
| PE anti-mouse CD5 antibody | rat | 53 - 7.3 | BD Bioscience | 553022 | 1:40 |
| FITC anti-mouse CD19 antibody | rat | 1D3 | BD Bioscience | 553785 | 1:40 |
| anti- human PD-L1 antibody | rabbit | - | Thermo Fisher Scientific |  | 1:200 |
| anti-human phospho-NF-kB p65 (Ser536) antibody | rabbit | 93H1 | Cell Signaling | 3033 | 1:100 |
| anti-phospo p44/42 MAPK (ERK1/2) (Thr202/Tyr204) antibody | rabbit |  | Cell Signaling | 9101 | 1:200 |
| anti Shc antibody | rabbit | A21236 | Merck Millipore | 06-203 | 1:1000 |
| neutralizing human PD-L1 antibody | rabbit | R639 | EliteRmab | 10084-R639 | 5 μg/ml |
| anti-CD3ζ antibody | mouse | 6B10.2 | Santa Cruz | sc-1239 | 1:100 |
| PE anti-human CD3ε antibody | mouse | OKT3 | BioLegend | 317308 | 1:100 |
| anti-p-Tyr antibody | mouse | 4G10 | Merck Millipore | 05-1050 | 1:100 |
| AF555 phalloidin | - | - | Invitrogen | A34055 | 1:100 |
| anti-GFP antibody | mouse | 3E6 | Invitrogen | A11120 | 1:200 |
| Anti-actin antibody | mouse | C4 | Merck Millipore | MAB1501 | 1:10000 |
| Alexa Fluor anti-mouse 488 secondary antibody | goat | - | Thermo Fisher Scientific | A11001 | 1:80 |
| Alexa Fluor anti-rabbit 555 antibody | goat | - | Thermo Fisher Scientific | A21428 | 1:80 |
| Alexa Fluor anti-rabbit 647 antibody | goat | - | Thermo Fisher Scientific | A21236 | 1:400 |
| Goat anti-Rabbit HRP antibody | goat | - | Jackson ImmunoResearch Laboratories | 11-035-144 | 1:20.000 |
| IgG2B, isotype control antibody | Rat | 141945 | R&D Systems | MAB0061 | 0.1 ng/ml |
| B-1a Cell Isolation Kit, mouse | - | - | Miltenyi Biotech | 130-097-413 | - |
| RosetteSep Human B Cell Enrichment Cocktail | - | - | StemCell Technologies | 15064 | - |
| RosetteSep Human CD8^+^ T Cell Enrichment Cocktail | - | - | StemCell Technologies | 15063 | - |
| Dynabeads Human T-activator CD3/CD28 | **-** | **-** | Gibco | 11132D | - |
| Recombinant human IL-2 | **-** | **-** | Miltenyi | 130-097-745 | 50 U/ml |
| Cell Tracker Blue | - | - | Thermo Fisher Scientific | C2110 | 10 μM |
| CFSE | - | - | Thermo Fisher Scientific | C34554 | 1.5 µM |
| Propidium Iodide | - | - | Sigma-Aldrich | 537059 | 0.5 µg/ml |
| Ca^2+^ ionophore A23187 | - | - | Sigma-Aldrich | C9400 | 1 µg/ml |
| CM-H_2_DCFDA | - | - | Invitrogen | C6827 | 5 µM |
| H_2_O_2_ | - | - | Merck Millipore | H1009 | 100 µM |
| IT-901 | - | - | R&D Systems | 5846 | 1 µM |

**Supplementary Table 2. List of the primers used in this study.**

| Gene | Forward 5’-3’ | Reverse 5’-3’ |
| --- | --- | --- |
| Human p66Shc | TCCGGAATGAGTCTCTGTCA | GAAGGAGCACAGGGTAGTGG |
| Human PD-L1 | GTACCTTGGCTTTGCCACAT | CCAACACCACAAGGAGGAGT |
| Human CD200 | GACCACGTCTGTTACCAGCA | GCCTTTGTTGACGGTTTGCT |
| Human CD270 | CCACCTCAATGGCCTAAGCA | TGGTCTGGTGCTGACATTCC |
| Human CD276 | GCAGCCTATGACATTCCCCC | GGTCCTCAGCTCCTGCATTC |
| Human HPRT1 | AGATGGTCAAGGTCGCAAG | GTATTCATTATAGTCAAGGGCATATC |
| Mouse p66Shc | TGAGTTGGGAGAGCAGAGGT | CTCATTCCGAAGTGGGTTGT |
| Mouse PD-L1 | TGCTGCATAATCAGCTACGG | GCTGGTCACATTGAGAAGCA |
| Mouse GAPDH | AACGACCCCTTCATTGAC | TCCACGACATACTCAGCAC |
